# Medical News and Miscellany

**Published:** 1897-10

**Authors:** 


### Medical News and Miscellany.

**Dr. C. M. Ramsdell,** has removed from Hagerman, N. M. to Lampasas, Texas, his old home.

---

**Dr. L. G. Thornton,** late of Columbus, Texas, has removed to Luling where he has formed a co-partnership with Dr. J. J. McCollum.

---

**Want to buy** a home and practice, with school and railroad facilities. Northern or Central Texas preferred. Address, P. O. Box, 542, Bonham, Texas.

---

**Dr. James E. Morris,** of Madisonville, son of our long time friend, Dr. John E. Morris, has gone to Louisville, Ky., to take a post-graduate course at the Medical Department, University of Louisville.

---

**Fine Location,** black land, railroad town, small competition, will induct purchaser into practice. Residence and office for sale. If you mean business, write for terms, etc. Address, "Juan" care of TEXAS MEDICAL JOURNAL.

---

**Medical Students** attending the Louisville schools are invited by the Y. M. C. A. of that city, to make themselves at home at the headquarters of the Association. The Secretary, Mr. Danner, requests the JOURNAL to say that an effort will be made to establish a branch in each college, and its members will be given privilege of gymnasium, baths, swimming pools, library, entertainments, etc., at reduced rates. For details address E. A. Forbes, 318 W. Broadway, Louisville, Ky.

**Hymeneal.**—We have received cards announcing the marriage of Dr. G. H. Wooten, of Austin, son of Dr. T. D. Wooten, President of Board of Regents, to Miss Ella Newsom, of McKinney, Texas, to take place at McKinney, on 5th of October, inst.

---

**Raised his Salary.**—The JOURNAL is informed that Dr. McLaughlin of this city, who has just been elected to the Chair of Practice, in the Texas Medical College, declined the offer at the salary paid his predecessor, $2,500. and that the Regents increased the pay to $3,000.

---

**Prof. J. F. Y. Paine, M. D.,** Professor of Gynecology and Dean in the Medical Department of the University of Texas, has resigned the deanship of the college despite the wishes and over the protests of the Regents. Prof. Allen J. Smith, M. D., Professor of Pathology and Microscopy, has been elected dean.

---

**The Chair of Physiology** in the Texas Medical College made vacant by the resignation (?) of Dr. Clopton, has been filled by the appointment by the Regents, of a young man by the name of Carter, assistant to the Chair of Physiology, University of Pennsylvania; a graduate of Johns-Hopkins University.

---

**The New York Polyclinic.**—We have for sale at a discount two orders for $50.00 each on this famous school. These orders can be presented and will be accepted in lieu of cash. To a physician who is going to take a post-graduate course during the coming fall or winter this is a chance to save some money. Address the TEXAS MEDICAL JOURNAL.

---

**Dr. J. A. Bodine,** who has been associated with Dr. John A. Wyeth, for the past five years, has located in San Antonio, where he expects to confine his practice to surgery. We know Dr. Bodine, and know that he is held in high esteem by Dr. Wyeth, and we bespeak for him a successful career in the Lone Star State.

---

**Our** esteemed contemporary of the *Northwestern Medical and Surgical Reporter*, Prof. Edgar Capps, M. D., Professor of Physiology and Lecturer on Diseases of the Brain in the Medican Department of the Fort Worth University at Fort Worth, Texas, is spending several months in New York City, taking special courses on the eye, ear, nose and throat under Professors Herman Knapp and May, at the New York Polyclinic and the College of Physicians and Surgeons. After some months of special preparation Dr. Capps will return to Fort Worth and will limit his practice exclusively to these branches of medicine.

